# Discontinuation of neoadjuvant therapy does not influence postoperative short-term outcomes in elderly patients (≥ 70 years) with resectable gastric cancer: a population-based study from the dutch upper gastrointestinal cancer audit (DUCA) data

**DOI:** 10.1007/s10120-024-01522-5

**Published:** 2024-06-26

**Authors:** Jingpu Wang, Zhouqiao Wu, Eline M. de Groot, Alexandre Challine, Nadia Haj Mohammad, Stella Mook, Lucas Goense, Jelle P. Ruurda, Richard van Hillegersberg

**Affiliations:** 1 Department of Surgery, University Medical Center Utrecht, Utrecht University, PO Box 85500, Utrecht, 3508 GA The Netherlands; 2 Key Laboratory of Carcinogenesis and Translational Research (Ministry of Education), Department of Gastrointestinal Surgery, Peking University Cancer Hospital and Institute, Beijing, China; 3 Department of Digestive Surgery, AP-HP, Hôpital Saint Antoine, 75012 Paris, France; 4 Department of Imaging and Cancer, Department of Medical Oncology, University Medical Center Utrecht, Utrecht University, Utrecht, The Netherlands; 5 Departments of Radiation Oncology, University Medical Center Utrecht, Utrecht University, Utrecht, The Netherlands

**Keywords:** Gastric cancer, Elderly patients, Neoadjuvant therapy, Short-term outcomes

## Abstract

**Background:**

For the elderly patients with gastric cancer, it may be more challenging to tolerate complete neoadjuvant therapy (NAT). The impact of discontinued NAT on the surgical safety and pathological outcomes of elderly patients with poor tolerance remains poorly understood.

**Methods:**

Gastric cancer patients received gastrectomy with curative intent from the Dutch upper GI cancer audit (DUCA) database were included in this study. The independent association of age with not initiating and discontinuation of NAT was assessed with restricted cubic splines (RCS). According to the RCS results, age ≥ 70 years was defined as elderly. Short-term postoperative outcomes and pathological results were compared between elderly patients who completed and discontinued NAT.

**Results:**

Between 2011- 2021, total of 3049 patients were included. The risk of not initiating NAT increased from 70 years. In 1954 (64%) patients receiving NAT, the risk of discontinuation increased from 55 years, reaching the peak around 74 years. In the elderly, discontinued NAT was not independently associated with worse 30-day mortality, overall complications, anastomotic leakage, re-intervention, and pathologic complete response, but was associated with a higher risk of R1/2 resection (*p*-value = 0.001), higher ypT stage (*p*-value = 0.004), ypN + (*p*-value = 0.008), and non-response ( p-value = 0.012).

**Conclusion:**

A decreased utilization of NAT has been observed in Dutch gastric cancer patients from 70 years due to old age considerations, possibly because of their high risk of discontinuation. Increasing the utilization of NAT may not adversely impact the surgical safety of gastric cancer population ≥ 70 years and may contribute to better pathological results.

## Introduction

Gastric cancer is the fifth most common cancer worldwide [1]. Gastrectomy is the primary curative treatment for gastric cancer patients [2, 3]. Numerous studies have demonstrated that multimodality treatment, which includes neoadjuvant therapy (NAT) or perioperative therapy plus surgery, can provide a better survival for patients with gastric cancer than surgery alone [4–7]. As most elderly patients have more comorbidities, worse physical performance, and a shorter life expectancy, there are concerns about the effectiveness and safety of NAT for these patients [8].

Due to the lack of substantial evidence to support the application of NAT in the elderly gastric cancer population that are not frail, the current medical field is inclined towards withholding this potentially effective treatment. On the other hand, aging may contribute to a portion of elderly gastric cancer patients having significantly declining organ function which might limit the ability to complete NAT. Whether discontinued NAT can still improve the pathological outcomes without adversely affecting the postoperative short-term outcomes in elderly patients with poor tolerance to NAT remain unclear. Some studies have shown that the toxicity of NAT was significantly associated with worse short-term postoperative outcomes in patients with gastric cancer [9–11]. Therefore, establishing evidence to guide the appropriate initiation of NAT is imperative to optimize patient outcomes and reduce avoidable adverse effects.

This study therefore aims to evaluate the current status of clinical application of NAT in patients with gastric cancer in the Netherlands by analyzing the association between age and the risk of not initiating and discontinuation of NAT. Also, the impact of discontinued NAT on short-term postoperative outcomes and pathological results in the elderly patients was investigated.

## Methods

The data of this population-based study were extracted from the DUCA database. DUCA is a mandatory national auditing registry for all hospitals performing esophageal and gastric cancer surgery in the Netherlands since 2011 [12, 13]. The DUCA includes patient and tumor characteristics, treatment details, short-term postoperative outcomes (up to 30 days after surgery), and histopathologic results [13–15]. In addition, multiple quality control measures are applied to verify and maintain quality of data in the database [15–18]. The scientific committee of DUCA approved this study, no further ethical approval or patient informed consent was required in accordance with Dutch laws.

### Patient selection

Gastric cancer patients who underwent surgery between 2011 and 2021 were included in this study. The exclusion criteria were defined as follows: Patients (1) with recurrent tumors; (2) younger than 18 years old; (3) without cT1-4aM0 tumors or cT stage; (4) underwent emergency surgery; (5) without curative gastrectomy; (6) without information on NAT; (7) without complication records.

### Variables

The variables extracted from DUCA database include sex, age, Charlson score, history of malignancy, history of thoracic or abdominal surgery, BMI, weight loss, type of NAT, 80% completion of planned NAT, year of surgery, emergency surgery, Lauren type, tumour location, cT stage, cN stage, ASA score, type of resection, curative intent, hospital volume, pT stage, pN stage, R0 resection, overall complications, anastomotic leakage, re-intervention (radiological/ endoscopic/ surgical), and 30-day mortality.

### Outcome measures

The primary outcome measure was 30-day mortality, and the secondary outcome measures included not initiating and discontinuation of NAT, overall complications, anastomotic leakage, re-intervention (radiological/ endoscopic/ surgical), R1/2 resection, ypT stage, ypN stage, and pathological complete response (pCR, defined as ypT0N0).

### Statistical analysis

The clinical characteristics of the included patients were described by using mean ± standard deviation (SD) or frequencies and percentages. The independent association between age and the risk of not initiating NAT in patients who received gastrectomy were described using multivariable logistic regression with visual 4-knots restricted cubic splines (RCS) model. In addition, the independent relationship between age and the risk of discontinued NAT (discontinued NAT was defined as completion of planned NAT less than 80%) in patients who underwent NAT plus gastrectomy was also described by using the multivariable logistic regression with visual 4-knots RCS model. Based on the results of the RCS models, patients beyond a specific age (with a reduction in the application of NAT from that age onward in the Netherlands) were defined as elderly patients, and only the elderly patients who underwent NAT plus gastrectomy were included in the following analyses. All the elderly gastric cancer patients were divided into discontinued NAT plus surgery (DNS) group and complete NAT plus surgery (CNS) group according to the completion of NAT (< 80% in DNS group, and ≥ 80% in CNS group). The clinical characteristics of the two groups were described by using mean ± standard deviation (SD) or frequencies and percentages and were compared using chi-square and ANOVA tests. Multiple imputation was used to impute missing values and generate 20 new datasets. Percentages and chi-square tests were used to describe and compare the 30-day mortality, postoperative morbidity, and re-intervention rates between the two groups. Multivariate logistic regression was used to compare the short-term postoperative outcomes and pathological results between the two groups, with the CNS group as the reference. A two-sided *p* < 0.05 was considered statistically significant. All statistical analyses were performed by using SPSS version 25.0 software (SPSS, Chicago, IL), R software version 4.1.3 and Graph Pad Prism version 8.0.

## Results

### Patient population

**Fig. 1:**
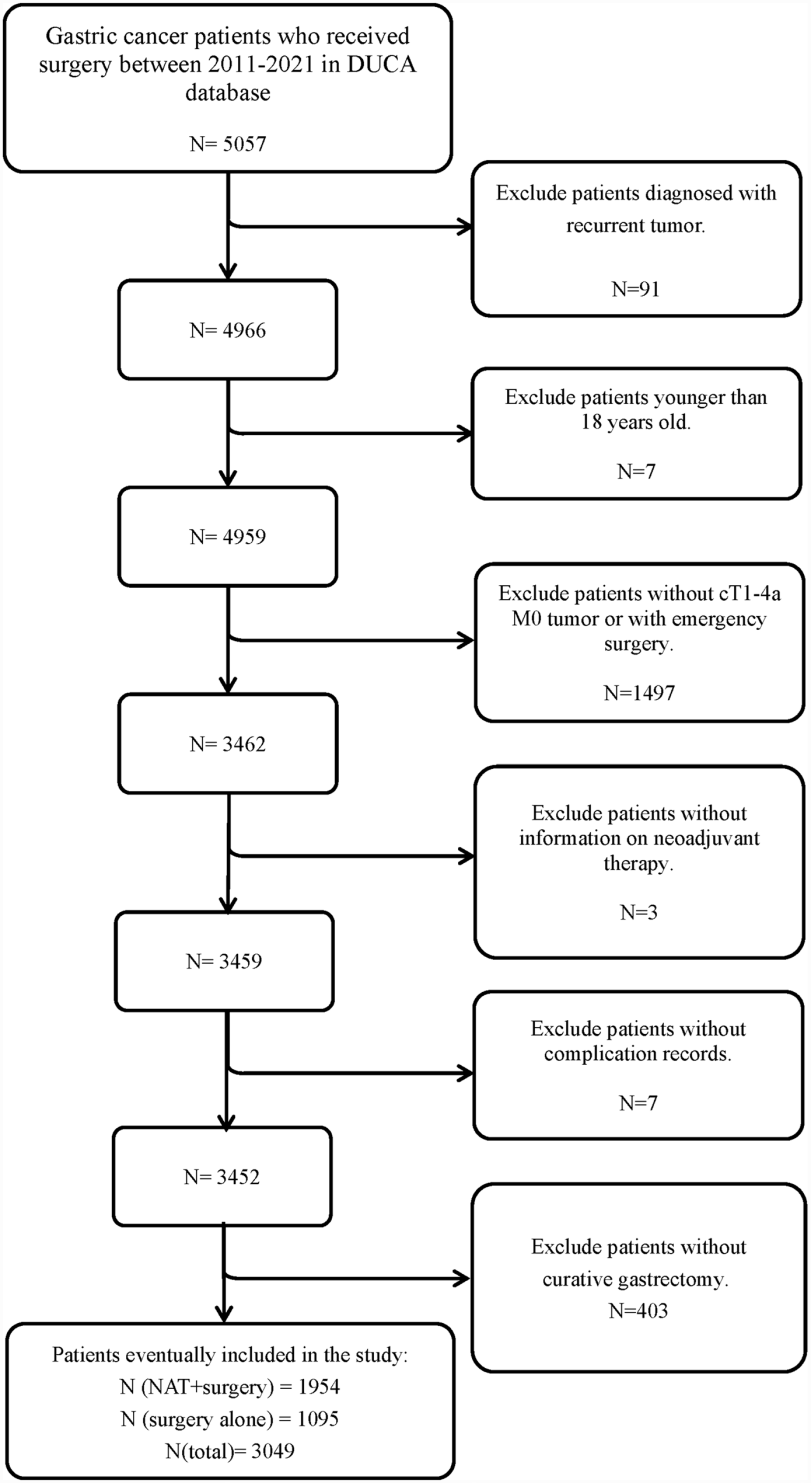
Patient selection process. DUCA, the Dutch upper GI cancer audit; NAT, neoadjuvant therapy

Between 2011 and 2021, 5057 gastric cancer patients were registered in the DUCA database, of whom 2008 patients were excluded. A total of 3049 patients who underwent gastrectomy for gastric cancer were included in this study (Fig. 1). The clinical characteristics of the included patients are shown in Table 1. Among the 3049 gastric cancer patients, 1095 (35.9%) underwent gastrectomy alone, while 1954 (64.1%) received NAT followed by gastrectomy. Of those who received NAT, 1858 (95.1%) received neoadjuvant chemotherapy, 87 (4.4%) received neoadjuvant chemoradiotherapy, and 9 (0.5%) were unknown. In addition, in 306 (15.7%) patients NAT was discontinued, 1606 (82.2%) completed NAT, and of 42 (2.1%) it was unknown.

**Table 1:** Clinical characteristics of all included resectable gastric cancer patients

Variables	All (3049)
Age	
18–64	945(31.0%)
65–69	462(15.2%)
70–74	543(17.8%)
75–79	562(18.4%)
80–84	406(13.3%)
85-	131(4.3%)
BMI	25.4 ± 4.4
Weight loss	4.9 ± 5.9
Sex	
Male	1903(62.4%)
Female	1146(37.6%)
Charlson score	
0	1308(42.9%)
1	729(23.9%)
2 +	1012(33.2%)
History of malignancy	
No	2552(83.7%)
Yes	480(15.7%)
Unknown	17(0.6%)
History of thoracic and abdominal surgery	
No	1842(60.4%)
Yes	1203(39.5%)
Unknown	4(0.1%)
Year of diagnosis	
2011–2013	642(21.1%)
2014–2017	1256(41.2%)
2018–2021	1151(37.8%)
Lauren type	
Intestinal type	1346(44.1%)
Diffuse type	898(29.5%)
Mixed type	179(5.9%)
Unknown	626(20.5%)
Tumor location	
Proximal 1 / 3	256(8.4%)
Middle 1/3	1013(33.2%)
Distal 1 / 3	1526(50.0%)
Whole stomach	140(4.6%)
Unknown	114(3.7%)
cT stage	
T1	217(7.1%)
T2	873(28.6%)
T3	1740(57.1%)
T4a	219(7.2%)
cN stage	
N0	1708(56.0%)
N1	842(27.6%)
N2	323(10.6%)
N3	59(1.9%)
Unknown	117(3.8%)
ASA score	
1	317(10.4%)
2	1671(54.8%)
3	998(32.7%)
4	45(1.5%)
Unknown	18(0.6%)
Neoadjuvant therapy	
No	1095(35.9%)
Yes	1954(64.1%)
Type of resection	
Total gastrectomy	1327(43.5%)
Partial gastrectomy	1722(56.5%)
Hospital volume	
< 20	826(27.1%)
20–40	1808(59.3%)
≥ 40	415(13.6%)

### The association between age and not initiating NAT

**Fig. 2:**
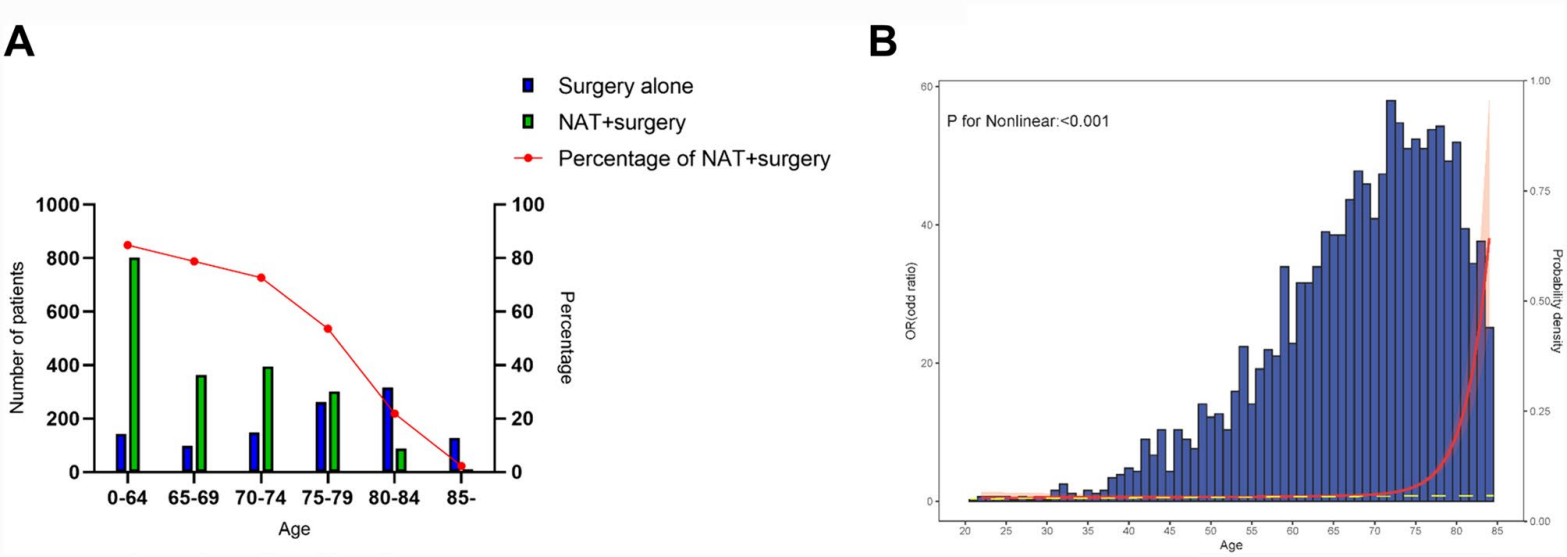
Number and proportion of patients with resectable gastric cancer treated with neoadjuvant therapy plus surgery for different age groups (**A**), and the independent association of age with the risk of not initiating neoadjuvant therapy (**B**). Adjusted variables included: sex, Charlson score, history of malignancy, history of thoracic or abdominal surgery, BMI, weight loss, year of surgery, Lauren type, tumor location, cT stage, cN stage, ASA score, and hospital volume. NAT, neoadjuvant therapy.

Trends in the proportion of patients who received NAT for different age groups are shown in Fig. 2A. The proportion of gastric cancer patients who received NAT decreased with age from < 65 years (802/945, 84.9%) to ≥ 85 years (3/131, 2.3%). The independent association between age and the risk of not initiating NAT in patients with gastric cancer is shown in Fig. 2B. The non-linear test showed a non-linear relationship between age and the risk of not initiating NAT (*p*-value: < 0.001), so that structural breakpoints could be identified from the RCS curve. From the age of 70 onwards, a decreased utilization of NAT has been observed in patients in the Netherlands, due to old age considerations. Therefore, patients ≥ 70 years old were defined as elderly patients in this study.

### The association between age and the discontinuation of NAT

**Fig. 3:**
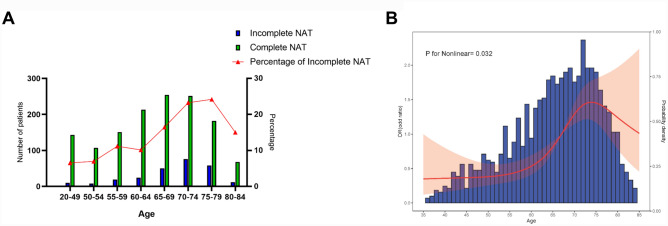
Number and proportion of patients in whom neoadjuvant therapy was discontinued among patients with gastric cancer who received neoadjuvant therapy plus surgery for different age groups (**A**), and the independent association of age with the risk of neoadjuvant therapy discontinuation (**B**). Adjusted variables included: sex, Charlson score, history of malignancy, history of thoracic or abdominal surgery, BMI, weight loss, year of surgery, Lauren type, tumor location, cT stage, cN stage, type of neoadjuvant therapy, ASA score, and hospital volume. NAT, neoadjuvant therapy

The trend of the proportion of discontinued NAT with increasing age in patients with resectable gastric cancer receiving NAT plus gastrectomy is shown in Fig. 3A. The proportion of discontinued NAT gradually increased from < 50 years (14/176, 8.0%), reaching a maximum (72/293, 24.6%) at the age of 75–79 years, and then decreased. The independent association between the risk of discontinued NAT and age is shown in Fig. 3B. The result of the non-linear test showed a non-linear relationship between the risk of discontinued NAT and age (*p*-value: 0.032), implying that the structural breakpoints could be identified from the RCS curve. The RCS analysis showed that the risk of discontinued NAT in gastric cancer patients receiving NAT plus gastrectomy was relatively stable before the age of 55, but started to increase from 55 years, reaching the peak around 74 years old.

### The impact of discontinued NAT on short-term outcomes in elderly gastric *cancer* patients (≥ 70 years)

Due to the fact that only three of the gastric cancer patients ≥ 85 years old received NAT plus gastrectomy, they were excluded. Ultimately, 768 elderly gastric cancer patients aged 70–84 who either completed or discontinued NAT were included in the following analyses. Out of them, 599(78.0%) patients completed NAT and in 169(22.0%) patients NAT was discontinued, the clinical characteristics of the two groups are shown in Table 2. Compared with the CNS group, the DNS group had more female (*p*-value = 0.049), worse ASA score (*p*-value = 0.038), and a higher proportion of hospital volume ≥ 40 (*p*-value = 0.049). There was no significant difference between the CNS group and DNS group in terms of 30-day mortality (3.5% vs. 3.6%, *p*-value = 0.958), the incidence of overall complications (37.2% vs. 34.9%, *p*-value = 0.224), anastomotic leakage (1.9% vs. 2.4%, *p*-value = 0.603), re-intervention rate (16.7% vs. 15.4%, *p*-value = 0.958), and pCR rate (9.0% vs. 4.8%, *p*-value = 0.079). However, the R1/2 rate (7.0% vs. 15.7%, *p*-value < 0.001) and the non-response rate (30.2% vs. 41.8%, *p*-value = 0.010) were higher in the DNS group. Multivariable logistic regression also demonstrated that there was no significant difference between the CNS group and DNS group in the risk of 30-day mortality, overall complications, anastomotic leakage, and re-interventions (Table 3). The multivariable logistic regression analysis based on pathological results showed that compared to the CNS group, the DNS group was associated with higher risk of R1/2 resection (OR: 2.90; 95%CI 1.54–5.45; *p*-value = 0.001), higher ypT stage (OR:1.85; 95%CI 1.22–2.80; *p*-value = 0.004), ypN + stage (OR:1.70; 95%CI 1.15–2.51; *p*-value = 0.008), and non-response (OR:1.69; 95%CI 1.12–2.56; *p*-value = 0.012), while there was no significant difference in the risk of pCR (OR:0.50; 95%CI 0.23–1.09; *p*-value = 0.080) between the two groups (Table 4).

**Table 2:** Clinical characteristics of patients aged 70–84 years with resectable gastric cancer (cT1-4a N0-3 M0) who underwent complete and discontinued neoadjuvant therapy plus surgery

Variables	All (768)	CNS group (599)	DNS group (169)	P-value
Age				0.103
70–74	388(50.5%)	303(50.6%)	85(50.3%)	
75–79	293(38.2%)	221(36.9%)	72(42.6)	
80–84	87(11.3%)	75(12.5%)	12(7.1%)	
BMI	25.4 ± 4.0	25.5 ± 4.0	25.2 ± 4.0	0.35
Weight loss	5.0 ± 5.5	4.8 ± 5.5	5.5 ± 5.2	0.207
Sex				**0.049**
Male	512(66.7%)	410(68.4%)	102(60.4%)	
Female	256(33.3%)	189(31.6%)	67(39.6%)	
Charlson score				0.96
0	302(39.3%)	234(39.1%)	68(40.2%)	
1	200(26.0%)	157(26.2%)	43(25.4%)	
2 +	266(34.6%)	208(34.7%)	58(34.3%)	
History of malignancy				0.088
No	625(81.4%)	483(80.6%)	142(84.0%)	
Yes	142(18.5%)	116(19.4%)	26(15.4%)	
Unknown	1(0.1%)	0(0%)	1(0.6%)	
History of thoracic and abdominal surgery				0.084
No	440(57.3%)	353(58.9%)	87(51.5%)	
Yes	328(42.7%)	246(41.1%)	82(48.5%)	
Year of diagnosis				0.491
2011–2013	121(15.8%)	90(15.0%)	31(18.3%)	
2014–2017	300(39.1%)	233(38.9%)	67(39.6%)	
2018–2021	347(45.2%)	276(46.1%)	71(42.0%)	
Lauren type				0.676
Intestinal type	382(49.7%)	291(48.6%)	91(53.8%)	
Diffuse type	190(24.7%)	152(25.4%)	38(22.5%)	
Mixed type	51(6.6%)	40(6.7%)	11(6.5%)	
Unknown	145(18.9%)	116(19.4%)	29(17.2%)	
Tumor location				0.419
Proximal 1 / 3	71(9.2%)	57(9.5%)	14(8.3%)	
Middle 1/3	290(37.8%)	231(38.6%)	59(34.9%)	
Distal 1 / 3	340(44.3%)	257(42.9%)	83(49.1%)	
Whole stomach	40(5.2%)	30(5.0%)	10(5.9%)	
Unknown	27(3.5%)	24(4.0%)	3(1.8%)	
cT stage				0.535
T1	14(1.8%)	10(1.7%)	4(2.4%)	
T2	211(27.5%)	167(27.9%)	44(26.0%)	
T3	476(62.0%)	374(62.4%)	102(60.4%)	
T4a	67(8.7%)	48(8.0%)	19(11.2%)	
cN stage				0.777
N0	400(52.1%)	311(51.9%)	89(52.7%)	
N1	233(30.3%)	181(30.2%)	52(30.8%)	
N2	97(12.6%)	75(12.5%)	22(13.0%)	
N3	12(1.6%)	9(1.5%)	3(1.8%)	
Unknown	26(3.4%)	23(3.8%)	3(1.8%)	
ASA score				**0.038**
1	45(5.9%)	36(6.0%)	9(5.3%)	
2	439(57.2%)	353(58.9%)	86(50.9%)	
3	272(35.4%)	203(33.9%)	69(40.8%)	
4	9(1.2%)	4(0.7%)	5(3.0%)	
Unknown	3(0.4%)	3(0.5%)	0(0%)	
Type of NAT				**0.003**
Chemotherapy	719(93.6%)	552(92.2%)	167(98.8%)	
Chemoradiotherapy	46(6.0%)	45(7.5%)	1(0.6%)	
Unknown	3(0.4%)	2(0.3%)	1(0.6%)	
Type of resection				0.343
Total gastrectomy	361(47.0%)	287(47.9%)	74(43.8%)	
Partial gastrectomy	407(53.0%)	312(52.1%)	95(56.2%)	
Hospital volume				**0.049**
< 20	174(22.7%)	140(23.4%)	34(20.1%)	
20–40	480(62.5%)	380(63.4%)	100(59.2%)	
≥ 40	114(14.8%)	79(13.2%)	35(20.7%)	

*CNS* complete neoadjuvant therapy plus surgery, *DNS* discontinued neoadjuvant therapy plus surgery

**Table 3:** Comparison of short-term postoperative outcomes between CNS group and DNS group in elderly patients (70–84 years) with resectable gastric cancer (cT1-4a N0-3 M0)

Group	OR (95%CI)	*p* value
30-day mortality		
CNS group (599)	Reference	
DNS group (169)	1.171 (0.422,3.250)	0.761
Overall complications		
CNS group (599)	Reference	
DNS group (169)	0.912 (0.624,1.333)	0.634
Anastomotic leakage		
CNS group (572)	Reference	
DNS group (166)	1.612 (0.450,5.768)	0.463
Re-intervention		
CNS group (599)	Reference	
DNS group (169)	0.996 (0.606,1.635)	0.986

Adjusted variables included: age, sex, Charlson score, history of malignancy, history of thoracic or abdominal surgery, BMI, weight loss, year of surgery, Lauren type, tumor location, cT stage, cN stage, type of neoadjuvant therapy, type of resection, hospital volume. CNS, complete neoadjuvant therapy plus surgery; DNS, discontinued neoadjuvant therapy plus surgery

**Table 4:** Comparison of pathological results between CNS group and DNS group in elderly patients (70–84 years) with resectable gastric cancer (cT1-4a N0-3 M0)

Group	OR(95%CI)	*p* value
R1/2 resection		
CNS (529)	Reference	
DNS (159)	2.895 (1.535,5.448)	**0.001**
Higher ypT stage (T0-2 Vs. T3-4)		
CNS (586)	Reference	
DNS (165)	1.851 (1.224,2.800)	**0.004**
Higher ypN stage (N0 Vs. N +)		
CNS (587)	Reference	
DNS (165)	1.697 (1.147,2.509)	**0.008**
Non-response		
CNS (546)	Reference	
DNS (134)	1.693 (1.122,2.555)	**0.012**
pCR		
CNS (587)	Reference	
DNS (166)	0.495 (0.225,1.087)	0.08

Adjusted variables included: history of malignancy, year of surgery, Lauren type, tumor location, cT stage, cN stage, type of neoadjuvant therapy, type of resection, hospital volume. CNS, complete neoadjuvant therapy plus surgery; DNS, discontinued neoadjuvant therapy plus surgery; pCR, pathological complete response

## Discussion

This population-based study showed that the risk of not initiating NAT due to old age increased after the age of 70 years in the Netherlands. A high risk of discontinued NAT was also observed after the age of 70 years. The discontinuation of NAT prior to gastrectomy in patients ≥ 70 years was not associated with higher risk of 30-day mortality, overall complications, anastomotic leakage, and re-intervention, but were associated with higher risk of R1/2 resections, higher ypT / N stage, and non-response compared with those who completed NAT plus gastrectomy. There was no significant difference in the risk of pCR between the two groups.

Results of this study showed that patients with resectable gastric cancer in the Netherlands were less frequently offered NAT due to old age considerations once they reached the age of 70. One of the reasons for this result might be the notion among healthcare providers that old age contributed to a decline in tolerability of NAT. The analysis of the association between the risk of discontinued NAT and age in this study supports this hypothesis, revealing a high proportion of discontinuation (169/768, 22.0%) even in selected patients older than 70 years. The decline in the functions of tissues and organs, particularly the hematopoietic function, caused by aging, may be a primary factor [19]. The results showed that the DNS group had worse ASA scores in the elderly, suggesting that worse ASA scores may be also associated with poorer organ or tissue function. Interestingly, the risk of discontinued NAT decreased after reaching a maximum at around 74 years old, which may be due to the fact that patients older than 75 years experienced even more stringent screening before NAT because of old age considerations.

Whether the increased risk of being unable to tolerate complete NAT should be a reason for reducing the application of NAT in the elderly is still unclear. First and foremost, it is essential to clarify whether NAT adversely affects the short-term postoperative outcomes of elderly patients who have difficulty tolerating NAT, although multiple published clinical trials have already demonstrated that NAT does not adversely affect the short-term postoperative outcomes of gastric cancer patients [4, 20–23]. In this study, a considerable proportion (169/768, 22.0%) of elderly patients were unable to complete NAT. The analysis of clinical characteristics showed that they had worse ASA scores compared with those who completed NAT. The worse ASA score may be an inherent feature of patients who cannot tolerate complete NAT, but as the ASA score was performed after NAT, it cannot be ruled out that it was caused by NAT. Therefore, the ASA score was not adjusted in the multivariate logistic regression. Nevertheless, the results indicated that the elderly patients who discontinued NAT could still achieve similar short-term postoperative outcomes as those who completed NAT. This seems to, to some extent, demonstrate that NAT does not impact the surgical safety of elderly patients with different tolerances. However, it is worth noting that the discontinuation of NAT may occur for various reasons, such as drug allergies, bone marrow suppression, patient willingness, etc. Whether different reasons for discontinuation will lead to a change in the conclusion is currently unclear.

As previously mentioned, NAT may not adversely affect the surgical safety of elderly patients with poor tolerance. However, analyzing the effectiveness of incomplete NAT in improving pathological results is equally crucial for refining the application strategy of NAT in elderly gastric cancer patients. The pathological results showed that the elderly patients who discontinued NAT had higher risk of higher ypT and ypN stage, R1/2 resection and non-response, which seems to be logical. These patients received fewer courses of NAT, so the effect of NAT on tumor downstaging likely became more limited. The higher risk of R1/2 resection of the elderly patients with discontinued NAT cannot rule out the possibility that more severe side effects restricted the extent of resection, but this cannot be proven based on the current data. Nevertheless, patients with gastric cancer who discontinued NAT still achieved a pCR rate similar to that of patients who completed NAT. This may be attributed to the limited impact of radiation or chemotherapy dosage on the treatment efficacy for gastric tumors that are sensitive to these therapies.

Based on the current evidence, it appears that utilizing NAT in elderly patients aged ≥ 70 years with gastric cancer does not adversely affect surgical safety, despite these patients’ generally poor tolerance to NAT. Additionally, as more elderly patients can receive complete or incomplete NAT, it may lead to improvement of pathological outcomes in the entire elderly surgical population, especially in terms of pCR. But caution should still be exercised in the application of NAT in the elderly. The results indicated that discontinuation of NAT was associated with a higher risk of non-response in elderly patients. For non-responders, NAT may not only fail to improve pathological outcomes but also cause toxic side effects and disease progression. Increasing the use of NAT in the elderly may raise the proportion of non-responders. Furthermore, as elderly patients with gastric cancer have a high risk of discontinuing NAT, and these frail patients have also been shown to have a high risk of losing surgical opportunities after NAT [24]. A study based on the Dutch population showed no difference in overall survival between gastric cancer patients ≥ 75 years who were treated with or without neoadjuvant chemotherapy [24]. One possible reason could be the inclusion of a considerable proportion of patients with limited life expectancy within the ≥ 75 years population. Limited life expectancy renders them less likely to benefit from more curative-intent cancer treatments in terms of survival. The elderly patients comprise a highly heterogeneous population. Therefore, pre-NAT comprehensive health assessment may be crucial for clinical decision-making regarding NAT in the elderly. It is not only necessary to identify patients who may lose the surgical opportunity after NAT, but also important to effectively identify patients with limited life expectancy who may not benefit from NAT on survival, thereby avoiding inappropriate use of NAT.

This study contains the following limitations: First, the number of gastric cancer patients older than 85 years who received NAT was quite limited, so they were not included in the primary analyses; Second, the patients who did not undergo surgery after NAT were not registered in the DUCA database. Third, the lack of long-term survival and quality of life has led to the inability to compare the effects of different treatment modalities on these measurements; Fourth, the data provided by DUCA lacks variables on the reasons for discontinued NAT and the number of courses and regimen of NAT received.

## Conclusion

A decreased utilization of NAT has been observed in patients aged 70 and older in the Netherlands due to old age considerations, possibly because of their high risk of NAT discontinuation. However, results of this study suggest that increasing the utilization of NAT may not compromise surgical safety. It could even potentially improve pathological outcomes in surgical gastric cancer patients aged 70 years and older.
